# Mitochondrial DNA maintenance in *Drosophila melanogaster*

**DOI:** 10.1042/BSR20211693

**Published:** 2022-11-10

**Authors:** Ana P.C. Rodrigues, Audrey C. Novaes, Grzegorz L. Ciesielski, Marcos T. Oliveira

**Affiliations:** 1 Departamento de Biotecnologia, Faculdade de Ciências Agrárias e Veterinárias, Universidade Estadual Paulista “Júlio de Mesquita Filho”, Jaboticabal, SP, Brazil; 2 Department of Chemistry, Auburn University at Montgomery, Montgomery, AL, U.S.A.

**Keywords:** DNA synthesis and repair, Drosophila melanogaster, mitochondria, nucleic acids

## Abstract

All 37 mitochondrial DNA (mtDNA)-encoded genes involved with oxidative phosphorylation and intramitochondrial protein synthesis, and several nuclear-encoded genes involved with mtDNA replication, transcription, repair and recombination are conserved between the fruit fly *Drosophila melanogaster* and mammals. This, in addition to its easy genetic tractability, has made *Drosophila* a useful model for our understanding of animal mtDNA maintenance and human mtDNA diseases. However, there are key differences between the *Drosophila* and mammalian systems that feature the diversity of mtDNA maintenance processes inside animal cells. Here, we review what is known about mtDNA maintenance in *Drosophila*, highlighting areas for which more research is warranted and providing a perspective preliminary *in silico* and *in vivo* analyses of the tissue specificity of mtDNA maintenance processes in this model organism. Our results suggest new roles (or the lack thereof) for well-known maintenance proteins, such as the helicase Twinkle and the accessory subunit of DNA polymerase γ, and for other *Drosophila* gene products that may even aid in shedding light on mtDNA maintenance in other animals. We hope to provide the reader some interesting paths that can be taken to help our community show how *Drosophila* may impact future mtDNA maintenance research.

## Introduction

**Figure 1:**
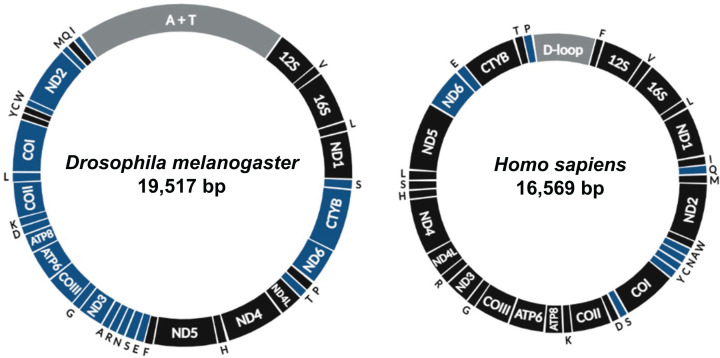
Schematic representation of the *Drosophila melanogaster* and human mitochondrial genomes The non-coding regions (A+T-rich region in flies, and D-loop in humans) are shown in gray. Genes encoded in the heavy and light strands (or their equivalents in the *Drosophila* mtDNA) are shown in black and blue, respectively. Transfer RNA genes are indicated by single-letter symbols, and the small and large ribosomal RNA genes by *12S* and *16S*, respectively. The genome size indicated is based on Lewis et al. [42] and Anderson et al. [130].

The mitochondrial DNA (mtDNA) in flies and humans is highly conserved in terms of gene content, as is in the vast majority of animal species, although genomic structure varies substantially (Figure 1). Its 37 genes encode 24 RNA components of the intramitochondrial protein synthesis machinery (22 transfer and 2 ribosomal) and 13 polypeptides of the oxidative phosphorylation (OXPHOS) system. Also conserved across metazoans is the fact that the mtDNA is maternally inherited (with very few exceptions), multicopy, circular and highly compacted, with all its gene content spanning between ∼15 and ∼20 kb, no intronic and very few and short intergenic regions (except for a major non-coding region containing *cis*-regulatory elements, see below for details) [1–3]. This genetic conservation clearly indicates a strong evolutionary pressure in animals to keep this set of 37 genes in a minute genome separate from the nuclear one, inside mitochondria. It is ordered in a way to perhaps optimize genomic transmission and maintenance, gene expression and, thus, mitochondrial function.

In humans, mtDNA mutations and defects in mtDNA maintenance are causes of diseases that occur in 1 in ∼5,000 individuals [4]. The problem is amplified by the fact that the multicopy nature of the mtDNA allows for pathogenic mutations to ‘hide’ in the population in 1 in every ∼200 carrier individuals [5]. The mixture of wild-type and mutated mtDNA molecules in a single individual is referred to as heteroplasmy, and is an integral part of our understanding of mtDNA diseases, as the proportion of mutated molecules in a cell/tissue type of a person dictates manifestation of disease symptoms [6,7]. Also essential for our understanding of mtDNA disease and biology are the processes of how mutations arise, which are often due to or associated with mtDNA maintenance defects. To date, a significant number of nuclear-encoded protein factors have been described in one or multiple processes involved with mtDNA replication, repair and transcription, and more are likely to be described in the future. Some of these function exclusively in mitochondria and are well documented, such as DNA polymerase γ (Pol γ) and the mtDNA helicase Twinkle, but the roles of other factors that are shared between the nuclear and mitochondrial proteomes certainly warrant further investigations.

Because of the conservation described above among animal mtDNA, the use of *Drosophila melanogaster* as a model organism to study mtDNA disease and biology in humans has been quite fruitful, and it still has the potential to provide more impactful findings as new technologies are constantly being developed for the fly [8–11]. Significant mechanistic differences in mtDNA maintenance have also been reported and are likely associated with the differences in mitochondrial genomic structure between flies and humans. Here, we review what is known about the mechanisms of mtDNA maintenance in flies, and provide a perspective on their tissue specificity and on the protein factors that should be further investigated in order for the scientific community to make the best use of this model.

## mtDNA replication in flies: the best studied maintenance process

DNA maintenance may be generally referred to as the set of DNA metabolic processes that guarantee the integrity and the proper number of copies of a genome. Here, we are considering as mtDNA maintenance processes all pathways involved in mtDNA replication and repair. We are also considering some RNA-related processes, since no dedicated primase has been found for this genome, and mtDNA transcription and transcript processing have a pivotal role in replication [3,12–16].

Replication of *D. melanogaster* mtDNA is by far the best studied of the maintenance processes. Analysis of replication intermediates using 2D native gel electrophoresis (2D-NAGE) showed in cultured cells and in whole animals at different developmental stages that a major replication mode operates [17]. Replication initiates and ends in the major noncoding region (Figure 1), also called the A+T-rich region (due to its unusually high deoxyadenylate and deoxythymidylate content, up to 96% [18]), proceeding unidirectionally, with coupled synthesis of the leading and lagging strands. In addition, it is evident that replication normally pauses at the two sites where the members of the mitochondrial transcription termination factor family mTTF and mTerf5 (encoded by the *mTTF* and *mTerf5* genes, respectively) bind the fly mtDNA [19]. Completion of replication is slow at the A+T-rich region, forming abundant four-way DNA junctions [17]. Indications for uncoupled leading and lagging strand synthesis have also been reported [17,20], but the prevalence of such a mechanism and the extent of the single-strandedness generated are still up for debate and deserve further investigations. Nevertheless, both the strand-coupled and -uncoupled replication modes appear to be conserved in metazoans and may be preferentially used in a particular physiological condition or tissue (see below for more discussion). Interestingly, an important replication mode described for vertebrates, the RITOLS (RNA incorporation throughout the lagging strand) [12,21,22], has not been detected in flies under normal physiology. Its existence in cultured S2 cells has been evidenced when the helicase Twinkle was overexpressed [23], but this still remains to be better characterized. We refer the reader to Ciesielski et al. [24] for a detailed review of the modes of mtDNA replication in animals.

As in mammals, the set of fly factors that function together at the replication fork to copy the mitochondrial genome, the so-called mtDNA replisome, is assumed to be only three. The helicase Twinkle translocates ahead of the fork on one DNA strand in the 5′-3′ direction, hydrolyzing nucleotide triphosphates and unwinding the parental double-stranded (ds) mtDNA into single-stranded (ss) DNA. Pol γ can then use this ssDNA as template to faithfully synthesize new mtDNA strands, a role that requires the balanced action of its 5′-3′ polymerase and 3′-5′ exonuclease enzymatic activities. The last replisome factor is the mitochondrial ssDNA-binding protein (mtSSB), which protects the released ssDNA from nucleolysis by wrapping it around its homotetrameric structure, resembling ‘the stitches on a baseball’. mtSSB also appears to serve as a hub to coordinate the functions of Twinkle and Pol γ [3,24–27].

Although mtDNA replisome proteins are significantly conserved at the amino acid sequence level and at least some similar modes of replication occur both in mammals and flies as discussed above, there are structural, functional and physiological differences between the mammalian and *Drosophila* mtDNA replisome proteins. This may reflect how differently organized mitochondrial genomes must be replicated *in vivo*. Perhaps, the most striking difference to our knowledge is in the structure and function of Pol γ. The fly enzyme has served as a model for our understanding of the Pol γ structure–function relationship for almost four decades (reviewed in [24,28]) and is likely the best studied polymerase in *Drosophila* [29]. In flies, as in most animals, this enzyme has two subunits: the catalytic subunit Pol γ-α (encoded by the *PolG1* gene, formerly *tamas*), which contains both the polymerase and exonuclease catalytic sites; and the accessory subunit Pol γ-β (encoded by the *PolG2* gene), which enhances enzyme processivity and fidelity [30–33]. Vertebrates have interestingly acquired a domain (H133-R182 in the human sequence) at the N-terminus of Pol γ-β (encoded by the *POLG2* gene in humans) that allows the accessory subunit to homodimerize [30,34,35]. It then makes more contacts with Pol γ-α (encoded by the *POLG* gene in humans) and perhaps grants a more refined regulation of Pol γ activities [36]. The importance of the dimeric Pol γ-β is such that there have already been several mutations in *POLG* and *POLG2* shown to disturb the Pol γ-β dimer or the interactions between Pol γ-α and the distal Pol γ-β subunit, and cause human diseases [37–39]. In addition, *in vitro* the trimeric (αβ_2_) human Pol γ can synthesize up to 5-fold more DNA than the dimeric (αβ) fly enzyme in a singly-primed template [40].

In the context of genome replication by the mtDNA replisome, however, this difference may not be as relevant, as *in vitro* DNA synthesis by the *Drosophila* Pol γ is highly (25- to 30-fold) stimulated by mtSSB (encoded by the *mtSSB* gene in flies, formerly *lopo*). On the other hand, mtSSB (encoded by the *SSBP1* gene in humans) stimulation of human Pol γ reaches only ∼6-fold, which makes the fly and human Pol γ-mtSSB systems functionally similar [40,41]. The ability to stimulate Pol γ *in vitro* appears to be partially related to the ability of mtSSB to organize the template DNA. DNA species formed by mtSSB binding that characterizes a denser template are more efficiently used by the human Pol γ, whereas *Drosophila* Pol γ is more active when mtSSB binding leads to more fully opened template DNA molecules, as visualized by electron microscopy [40]. Interestingly, vertebrate mtSSBs, including the human protein, have an extension of loop 2,3 (S51-L59 in the human sequence) which contributes to the opening of the template DNA and to the overall stimulation of DNA synthesis by human Pol γ [27,40]. As in other invertebrates, *Drosophila* mtSSB has a short loop 2,3, and its deletion does not appear to interfere with template DNA conformation and stimulation of the fly Pol γ *in vitro* [40], although the expression of a mutant mtSSB lacking loop 2,3 causes mtDNA depletion in *Drosophila* S2 cells in culture [27]. We speculate that these differences are related to the different organizations of the mitochondrial genomes in humans and *Drosophila*, especially the fact that the fly mtDNA is highly A+T rich (∼82%, [42]) and would be a template thermodynamically easier to unwind and replicate.

Another interesting fact about the Pol γ-mtSSB system is that, in humans, *in vitro* physical interactions between these two proteins appear to occur more prominently in the absence of the Pol γ-β dimer [43]. In addition, human Pol γ-α alone *in vitro* is an efficient gap-filling polymerase and in fact can participate in mtDNA repair [28]. The α and β subunits of the *Drosophila* Pol γ are relatively unstable individually in solution, unlike the human proteins that can be purified independently and assembled *in vitro* [45,46]. This would argue that the *Drosophila* subunits are more often found complexed forming the holoenzyme inside mitochondria and that interactions between mtSSB and Pol γ-α in the absence of Pol γ-β are less likely. This is consistent with the data on the relative transcript abundance of the Pol γ catalytic and accessory subunits that we present below (see next sections), indicating >5-fold excess of the latter.

*In vivo*, a remarkable difference between mammalian and *Drosophila* Pol γ functions is related to the enzyme’s exonuclease activity. The abolishment of such activity by alanine substitution of a catalytic aspartate in the conserved Exo II motif of Pol γ-α (D257 in mice, D263 in flies) created ‘mtDNA-mutator’ animals. Since the enzyme is no longer able to proofread newly synthesized DNA, these animals indeed accumulate mtDNA mutations (nucleotide substitutions and deletions) [47,48]. A collection of age-related phenotypes clearly established premature aging as the main feature of the mtDNA-mutator mouse, whereas for the mtDNA-mutator fly, developmental lethality at the late larval or early pupal stages with no apparent changes in mitochondrial respiration rates was the prominent outcome. Two types of mtDNA mutations found in the mutator fly are worth discussing, as they may relate to the biology of *Drosophila* mtDNA. First, the number of accumulated A-to-T transversions (18% of total point mutations) were almost as high as the A-to-G or T-to-C (∼25% each) transitions, and curiously no C-to-G transversions were detected [47]. The accumulation of deoxyadenylates and deoxythymidylates in insect mitochondrial genomes is thought to be a product of selection pressure operating on the mtDNA molecules themselves, as A+T-richer molecules would have the advantage of being unwound more easily during mtDNA replication and transcription in organisms with very fast metabolism that require fast DNA metabolic processes [18,49]. Such pressure would have to be quite strong for A-to-T substitutions to be detected at levels this high even before development is completed. Alternatively, we speculate that the A+T-richness of insect/*Drosophila* mtDNA may at least in part be explained by biased nucleotide incorporation by Pol γ *in vivo*, which indicates that the selection pressure to keep high A+T-rich mitochondrial genomes may be influencing the enzyme’s nucleotide selectivity. Interesting insights into this matter may be provided in the future by two parallel experimental approaches: (1) analyses of the deoxyribonucleotide pools inside the fly mitochondria and their control by particular gene products; and (2) molecular evolutionary analysis and structural modeling with focus on how the fingers subdomain of Pol γ-α may interact with the polymerase catalytic site in the palm subdomain. This will help us understand how mitochondrial genomes in humans and flies are differently shaped.

The second type of noteworthy mutation in the mtDNA-mutator fly is the accumulation of linear mtDNA molecules with deletions spanning almost the entire A+T-rich region. Although deleted, linear mtDNA is also present in the mtDNA-mutator mouse [50], these molecules in the mutator fly appear to be the only type of mutation clearly associated with the lethality caused by the lack of Pol γ’s exonucleolytic capacity at the onset of metamorphosis. The point mutations in general are associated only with developmental delay [47]. The extensive tissue rearrangements that occur during metamorphosis are initiated with programmed cell death and histolysis of most larval organs, which allows the biogenesis of the adult organs as the pupal stage progresses. Metabolically, there must be a significant change in mitochondrial function during this period, as the organism transitions from an efficient feeder and biomass accumulator to an efficient flying ‘athlete’. In the former, mitochondria act as the main anabolic hub in the cells, whereas in the latter these organelles (at least in the thoracic flight muscles) are committed to the complete oxidation of fuels for bulk ATP production via OXPHOS. We speculate that these significant changes in metabolism may be accompanied by important alterations in the mtDNA maintenance processes in one or more main *Drosophila* tissues, a feature that may be required in holometabolous insects and dependent on Pol γ’s exonucleolytic activity but absent in mammals. A tissue-specific role for Pol γ in *D. melanogaster* has already been shown in mtDNA elimination during spermatogenesis, a mechanism that contributes to the uniparental inheritance of this genome, and that is surprisingly independent of the enzyme’s exonucleolytic activity [51]. We discuss more about the possible tissue specificity of mtDNA maintenance processes below.

Twinkle (encoded by the *mtDNA-helicase* gene in *Drosophila*), our last mtDNA replisome factor, is a homohexameric/heptameric ring-shaped helicase, homologue of the bacteriophage T7 bifunctional gp4 [35,52]. The viral enzyme has a C-terminal domain that is highly conserved with Twinkle sequences from all eukaryotes [53], responsible for helicase activity *per se*. Also conserved is the Linker region domain, which is predominantly involved with subunit interactions and the stability of the hexamer/heptamer [54]. Interestingly, the N-terminal primase domain of gp4 synthesizes RNA primers for Okazaki fragment synthesis at the T7 replication fork [55], a function that is not conserved in the human (encoded by the *TWNK* gene, formerly *C10orf2*) or fly Twinkle [54,56]. It is easier to understand why Twinkle in humans and other vertebrates lack primase activity: this domain has diverged significantly in amino acid sequence [53], although structurally we have shown that part of it may still fold into an RNA polymerase-like structure [54] that allows for DNA binding [56]. However, in *D. melanogaster* and other invertebrates, Twinkle’s N-terminal domain has several of the conserved motifs that are functionally important for primase activity in the T7 gp4, including four cysteines that coordinate the binding of a zinc atom in motif I [57]. Remarkably, these cysteines in the fly enzyme (C68, C71, C102, and C105) are used for interactions with an iron-sulfur (2Fe-2S) cluster [58]. The zinc-binding motif in the T7 gp4 is important to help the primase domain interact with the template DNA strand and deliver the RNA primer to the T7 DNA polymerase [55,59]. To date, the only function associated with the homologous 2Fe-2S cluster-binding motif in *Drosophila* Twinkle is general protein stability *in vitro*, which may reflect as enhanced helicase activity *in vivo* [58,60]. In S2 cells cultured under standard conditions, expression of Twinkle lacking the 2Fe-2S cluster did not affect cell viability or mtDNA copy number [44], but it remains possible that this prosthetic group influences Twinkle stability and function only under certain conditions or in specific tissues. Because iron–sulfur clusters are redox active protein cofactors and are present in many nuclear DNA metabolic enzymes (reviewed in [61]), mtDNA maintenance in flies may be modulated by the oxidative state inside mitochondria via Twinkle. In fact, it has been shown that increased mitochondrial reactive oxygen species (ROS) produced as a consequence of elevated mitochondrial membrane potential leads to mtDNA depletion in S2 cells [62]. It is tempting to consider the 2Fe-2S-bearing Twinkle as one of the effectors of such mtDNA phenotype, but this phenomenon warrants further investigation, especially in whole fly tissues.

Recently, we have shown that overexpression of Twinkle in *Drosophila* S2 cells causes mtDNA deletions [23], creating a model for the studies of these that are the most frequent type of mutations in human mtDNA (reviewed in [26]). The deletions occur specifically at the fly’s A+T-rich region, which loses several of its internal tandem repeats, remaining apparently functional and sustaining mtDNA replication and transcription for as long as a year [35]. This is associated with an increase in four-way junction replication intermediates that map at the A+T-rich region and excessive catenation of mtDNA molecules, indicating that an increase in Twinkle levels might disturb replication termination [23]. Interestingly, expression of the exonuclease-deficient Pol γ-α in the mutator fly also generates deletions of the A+T-rich region (with linear mtDNA molecules [47]), suggesting that this is an especially sensitive region, prone to genomic rearrangements. Accordingly, we have shown that the number of tandem repeats in the mtDNA A+T-rich region is quite variable among fly lines originated from different geographic locations, and that in general mtDNA copy number is higher in flies bearing mitochondrial genomes with more A+T tandem repeats [63]. More mtDNA also correlated with longer developmental times, which in the wild may impact organismal fitness negatively as faster development can grant individuals an advantage for the limited food resources available during the larval stages [63–66]. Therefore, unlike in mammals, for which mtDNA deletions always encompass the coding region (with loss of several protein- and transfer RNA-coding genes; see [26] for details) with clear negative outcomes for mitochondrial function, the occurrence of deletions in *Drosophila* mtDNA appear to frequently involve the non-coding A+T-rich region and have either positive or negative effects, which still need further investigation.

## Beyond the mtDNA replisome, but not really

Overexpression of Twinkle in cultured S2 cells and in flies also increases mtDNA copy number 30–40% [23,35,67,68]. In S2 cells, this is associated with a significant drop in replication pause intermediates at the two mTTF/mTerf5 binding sites in the mtDNA [23], indicating that these pauses are regulated and prevent excessive mtDNA copy number. Interestingly, overexpression of *Dm*NUBPL (encoded by the *CG3262* gene), the likely 2Fe-2S cluster donor for the *Drosophila* Twinkle, also alleviates the same replication pause sites in the mitochondrial genome [60]. Knockdown of mTTF has a similar effect on the abundance of these replication pause intermediates, except that in this case mtDNA transcription termination is affected, leading to random replication fork stalling, impaired lagging strand synthesis, and mtDNA depletion, likely due to unregulated collisions between the mtDNA replisome and the transcription machinery [19]. Knockdown of mTerf5 led to the opposite molecular effect, with enriched replication pausing at mTTF/mTerf5 binding sites, but to the same phenotypic effect as mTTF knockdown: extension of the larval stage, with prevention of the start of the metamorphosis stage (although the larvae do reach the critical weight that would allow them to do so) and ultimately death [19]. Interestingly, this is the same developmental arrest phenotype observed for flies that ubiquitously overexpress Twinkle alleles with severe mutations, such as alanine substitution of a catalytic lysine (K388A in flies; K421A in humans) and substitutions associated with human progressive external ophthalmoplegia (W441C and A442P in flies; W474C and A475P in humans) [67,68]. This phenotype is also observed for flies with ubiquitous knockdown of one of the core components of the mitochondrial transcription machinery, the transcription factor B2 (mtTFB2, encoded by the *mtTFB2* gene in flies, and by the *TFB2M* gene in humans) [69].

The binding sites for the mTTF/mTerf5 complex in the *Drosophila* mtDNA are also the regions mostly affected by misexpression of RNase H1 (encoded by the *rnh1* gene in flies, and by the *RNASEH1* gene in humans), which is likely the main enzyme that processes mitochondrial transcripts to be used as primers for mtDNA replication [70]. RNase H1 overexpression, like Twinkle overexpression and mTTF knockdown, led to decreased replication pausing at the mTTF/mTerf5 binding sites, whereas its knockdown increased pausing similarly to mTerf5 knockdown [13]. In addition to the A+T-rich region, the mTTF/mTerf5 binding sites of the fly mitochondrial genome are clearly important *cis*-regulatory regions, in which several protein factors may function to coordinate replication and transcription, with impact in mtDNA maintenance. And as for the A+T-rich region, because the mTTF/mTerf5 binding sites are possible regions of constant conflict between the mtDNA replisome and the transcription machinery, it is expected that they are also genomic regions prone to rearrangements. In fact, upon selection for mtDNA recombinants in *Drosophila*, Ma & O’Farrell [71] observed that the recombinant molecules frequently had crossover sites very close to the mTTF/mTerf5 binding sites.

Although recombination has been better recognized in recent years [72], it is still evident this is not a major mechanism that contributes to inherited mtDNA variability in most animals [71,73]. Nevertheless, as part of a ‘formerly neglected, but now well-recognized’ DNA repair system, mtDNA homologous recombination and non-homologous end-joining seem efficient in mammalian cells, especially when this genome is inflicted with double-strand breaks [74,75]. In *Drosophila*, double-strand breaks also enhance the selection for individuals with recombinant mtDNA molecules [71]. At least in cultured S2 cells, this double-strand break recombinational repair mechanism is as rapid and effective as in the nucleus [76]. In addition, there is now evidence showing that the deletions observed in the human and fly mtDNAs are caused by a particular type of recombination event called copy-choice recombination [23,26], which can occur with the sole presence of the Pol γ holoenzyme in *in vitro* assays containing a DNA template with direct repeats [77].

Base excision repair (BER) is the best studied of all ‘formerly neglected, but now well-recognized’ mtDNA repair systems, primarily because of its capacity to eliminate oxidative damage caused by mitochondrial ROS [78]. To our knowledge, information on mitochondrial BER in flies is extremely poor in the literature, although this organism has contributed significantly to our understanding of DNA repair in general [79]. The *Drosophila* homolog of 8-oxoguanine DNA glycosylase Ogg1 (encoded by the *Ogg1* gene) appears to contain an N-terminal mitochondrial targeting sequence, but a loss-of-function allele of *Ogg1* did not increase somatic mtDNA mutations in oxidatively stressed flies [80]. In addition, overexpression of a mitochondrially targeted Ogg1 and another DNA glycosylase, RpS3 (encoded by the *RpS3* gene), in S2 cells lowered the levels of 8-oxoguanine in the mtDNA, but it also increased susceptibility to oxidative stress, which is likely the result of accumulation of toxic BER intermediate molecules [81]. Using a biochemical approach, Garreau-Balandier et al. [82] surprisingly showed that mitochondrial extracts from whole male individuals lack the capacity to excise 8-oxoguanine paired with deoxycytidylate or deoxyadenylate, which may explain the odd results above with misexpression of Ogg1. *Drosophila* mitochondria were actually quite efficient in removing other products of DNA damage: abasic sites, uracil paired with deoxyguanylate, 1,*N*^6^-ethenoadenine paired with deoxythymidylate, and thymine glycol paired with deoxyadenylate. Given the high A+T richness of the fly mtDNA, it is expected that upon excessive ROS, 1,*N*^6^-ethenoadenine and thymine glycol would be found more abundantly in this genome and constitute a more ‘urgent problem” to mitochondria than 8-oxoguanine. We speculate that these results reflect the biology of the A+T-rich mitochondrial genomes in insects, and that studying 8-oxoguanine accumulation and excision in *Drosophila* mtDNA as a model for understanding base oxidation and BER in mammalian mitochondria may not be a fruitful research path.

Given the lack of more information on mtDNA repair in flies, we decided to use the data provided by Allkanjari and Baldock [83] on the enriched DNA repair proteins found in the human mitochondrial proteome to generate a list of *Drosophila* homologs and possibly a rough overview for what mtDNA repair looks like in this organism. It is important to stress that this approach does not provide evidence whatsoever that the gene products of the fly homologs we found are also located in mitochondria, but we believe this may help guide future research. Supplementary Table S1 shows that *Drosophila* lacks approximately a third of all mtDNA repair genes found in humans, consistent with a recent review on the lack of several nuclear DNA repair genes [79]. Interestingly, some of these genes are conserved in organisms as diverse as bacteria and plants, indicating that flies and other holometabolous insects have lost them. One example is *UNG*, which in humans encodes a mitochondrial and a nuclear version of uracil–DNA glycosylase, responsible for eliminating uracil from DNA molecules that may arise from cytosine deamination [84]. The lack of a *UNG* homolog and the absence of dUTPase (encoded by the *dUTPase* gene in flies, and the *DUT* gene in humans) expression in *Drosophila* larvae has been linked to the accumulation of uracil in the nuclear genomic DNA, which appears to be part of normal developmental physiology [85]. Considering that uracil-DNA glycosylase activity has been shown in protein extracts from adult fly mitochondria and nuclei [82], additional factor(s) with this enzymatic activity must be present in this organism. Nevertheless, it is not yet clear whether fly mtDNA also accumulates uracil. We speculate it very likely does and that there may not be a strong pressure for the removal of this type of DNA ‘damage’, since replication of a template containing deoxyuridylate would allow incorporation of a deoxyadenylate in the newly-synthesized mtDNA strand, promoting higher A+T content. It is clear, though, that mtDNA repair in *Drosophila* is still an area wide open for new discoveries.

## What can omics data reveal about the tissue-specificity of *Drosophila* mtDNA maintenance?

Maintenance of the mitochondrial genome, especially the processes of replication and transcription, is often depicted in the literature very statically. This is best exemplified by how the mtDNA replisome is portrayed, as ‘the’ machinery responsible for copying this genome. Twinkle, Pol γ and mtSSB are indeed evolutionary (except for mtSSB) and functional equivalents of the helicase, polymerase and single-stranded DNA-binding protein of the well-known bacteriophage T7 replisome [86], but significantly less has been revealed to date about the physical and mechanistic interactions among the mtDNA replisome components. The static acceptance of the mtDNA replisome contrasts the fact that diverse maintenance mechanisms have constantly been described in the literature in the last two decades (reviewed in [24]), some of which appear to preferentially occur in a tissue-specific manner. For example in the mouse, Goffart and co-workers have recently shown that the mtDNA maintenance mechanisms operating in tissues with high OXPHOS activity are distinct from those in other tissues, so that mtDNA integrity, copy number, and gene expression are kept optimal even upon the constant insults promoted by ROS [87]. In muscular and neuronal tissues, increased levels of damaged mtDNA molecules have been observed along with abundant four-way DNA junctions, indicating that recombination may be the main maintenance mechanism in these tissues. Interestingly in *Drosophila*, we have shown that mtDNA copy number in whole adult flies is negatively correlated with levels of mitochondrial oxygen consumption and OXPHOS supercomplexes [63], the latter being considered a mechanism to improve electron transfer efficiency and lower ROS production [88]. Conversely, as mentioned above, mtDNA copy number drops in *Drosophila* S2 cells with elevated mitochondrial membrane potential and ROS [62]. Once again, ROS may play a fundamental role in determining how mtDNA is maintained in flies, as well as in mammals.

Although it is yet to be shown how copy number is potentially regulated by mitochondrial ROS in flies, the general variability in mtDNA maintenance mechanisms in animal tissues invokes that: (1) other unknown factors may also play important roles, likely with tissue specificity; and/or (2) the mtDNA replisome components may have additional activities. The primase-polymerase PrimPol (encoded by the *PRIMPOL* gene in humans) is a good example of a recently identified new player: it functions in a mechanism that rescues stalled replication forks [89], which if not resolved, can result in double-strand breaks and cause mtDNA depletion or deletions [90]. Curiously, the *D. melanogaster* genome does not encode a PrimPol homologue ([29] and Supplementary Table S1), indicating that the bypass of mtDNA replication problems in this model organism is accomplished through distinct mechanism(s). Additional roles for the mtDNA replisome factors is exemplified by the more recently identified strand-annealing, strand-exchange and branch migration activities of the human Twinkle *in vitro*, which indicates it also participates in recombinational repair [91,92]. These new catalytic activities are yet to be shown for the *Drosophila* enzyme, which as discussed above, is quite distinct from its human homologue due to the presence of a 2Fe-2S cluster.

Here, we decided to take advantage of the abundant tissue-specific transcriptomic data publicly available for *Drosophila* and seek expression patterns that may indicate distinct mtDNA maintenance mechanisms. We believe this may be of particular relevance since large-scale RNAi screening in *Drosophila* S2 cells for mtDNA copy number regulators, in addition to confirming the roles of known factors (see Supplementary Table S2), appears to have primarily revealed processes that are secondary to nucleic acid metabolism, such as cytoplasmic protein synthesis and degradation, vesicle trafficking, and maintenance of mitochondrial membrane potential [62]. Interestingly, this study failed to identify the mitochondrial Lon protease (encoded by the *Lon* gene) as a factor involved with another secondary effect on mtDNA copy number: the degradation of TFAM (encoded by the *TFAM* gene), which is likely the most important protein for mtDNA compaction and is also involved with transcription [93]. Nonetheless, we realize that analyses of transcript levels like the ones we are presenting here can hardly be used to express protein levels or enzyme function, and do not necessarily reflect maintenance mechanisms. Therefore, our data should be seen more as a hypothesis-generating approach, that we place as perspective and hope can guide future research in this area. We included in these analyses only data from factors whose roles in fly mitochondria are better known, or for which there is strong evidence that its homolog functions in human/mammalian mitochondria. These include 21 proteins involved with replication, transcription, DNA/RNA processing, DNA packaging, and only a few of the repair proteins listed in Supplementary Table S1 (see Supplementary Table S2 for complete list). We also realize that our analyses may be skewed because transcript levels from genes in our list whose products also localize to the nucleus, such as *Fen1*, *DNA2*, *rnh1* and the topoisomerases, may be mostly influenced by their nuclear function, but we still hope to observe patterns worth being pursued experimentally in the future.

**Figure 2:**
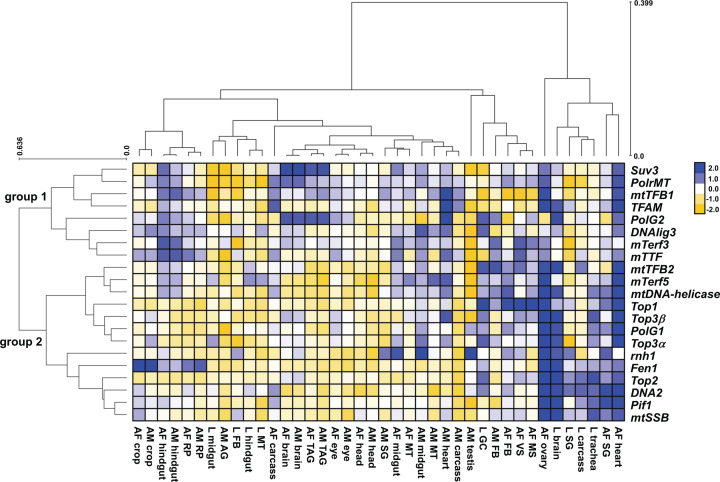
Hierarchical cluster analysis of the relative expression levels of *Drosophila* mtDNA maintenance genes across different fly tissues Transcript levels were obtained from FlyAtlas 2 [95], processed as described in the Methods, and shown in the heatmap as log_2_(fold change). The analysis was performed using EXPANDER [126]. L, AM, and AF indicate larval, adult male and female, respectively. The tissues sampled were: head, brain, eye, trachea, salivary gland (SG), crop, heart, midgut, hindgut, malpighian tubules (MT), garland cells (GC), rectal pad (RP), thoracicoabdominal ganglion (TAG), fat body (FB), carcass, ovary, virgin spermatheca (VS), mated spermatheca (MS), testis and accessory glands (AG). Groups 1 and 2 indicate respectively the ‘transcription/transcript processing’ and ‘replication’ clusters of genes formed with the analysis. See text for more details.

We collected transcript data from 40 distinct fly tissues from different developmental stages and sex, deposited in the databank of the FlyAtlas 2 project (www.flyatlas2.org, [94,95]), and first performed a hierarchical cluster analysis to reveal coexpression patterns. Figure 2 shows that our mtDNA maintenance genes form two groups: group 1 is represented by *PolrMT*, which encodes the mitochondrial RNA polymerase, and by other mitochondrial transcription-related factors; group 2 contains *PolG1*, *mtDNA-helicase* and mostly other replication factors. Genes in group 1 are characterized in general by higher expression in the adult (males and females) hindgut, midgut, brain and thoracicoabdominal ganglion, and in the adult male heart. Group 2 genes are typically expressed at relatively low levels, except in larval brain, trachea and carcass (which is mostly muscle tissue), and in the salivary gland of larvae and adult females. The heart and the ovaries of adult females present strong expression of almost all genes, regardless of group, whereas the larval hindgut, midgut, fat body and Malpighian tubule, the adult male testis and accessory gland, and the adult (males and females) head had low transcript levels of almost all genes (Figure 2). The differences in expression pattern between groups 1 and 2 are not explained by correlations with the expression of selected OXPHOS and other mitochondrial proteins, as groups 1 and 2 cluster to form a mitochondrial nucleic acid metabolism group (Supplementary Figure S1).

**Figure 3:**
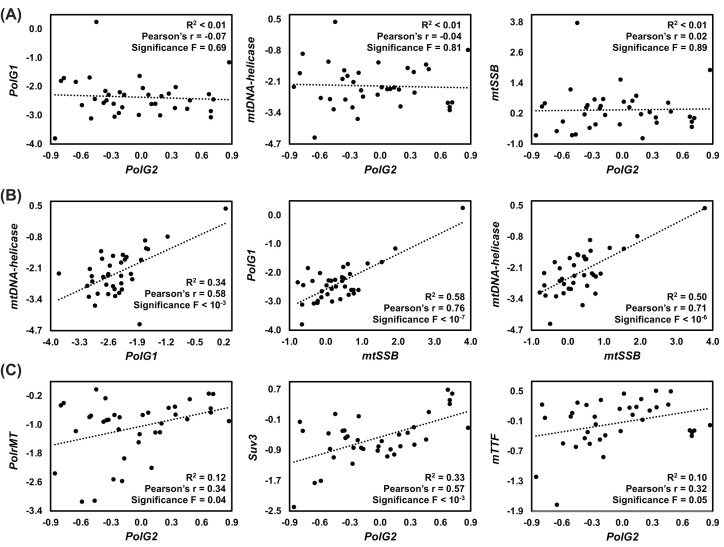
*PolG2* expression across *Drosophila* tissues correlates with mitochondrial RNA metabolism, and not mtDNA replisome genes Lack of significant correlations between *PolG2* transcript levels and those of the indicated mtDNA replisome genes is shown in (**A**). Significant correlations among the transcript levels of the other mtDNA replisome genes, and between *PolG2* transcript levels and those of the indicated mitochondrial RNA metabolism genes are shown, respectively, in (**B**,**C**). Transcript levels were obtained from FlyAtlas 2 [95] and were processed as described in the Methods. The *R*^2^, Pearson’s *r*, and the significance *F* values were obtained from linear regression analyses performed using Microsoft Excel. Tissues from which expression levels were calculated are listed in Figure 2.

Perhaps the most striking finding in our data is the separation of *PolG2* (placed in group 1 with mostly transcription-related factors) from the other mtDNA replisome genes (group 2). This is also very apparent in a principal component analysis (PCA) of the tissue-specific transcript data (Supplementary Figure S2). When *PolG2* transcripts were analyzed in pairwise comparisons with *PolG1*, mtDNA-helicase and *mtSSB* transcript levels, no significant correlations were found (Figure 3A). *PolG2* transcript levels in fact correlated significantly with those of mitochondrial RNA metabolism genes, such as *PolrMT*, *mTTF* and *Suv3* (Figure 3C). This pattern of expression is also observed in the tissue-specific transcriptomic data from the modENCODE project (http://www.modencode.org/, [96]), which is deposited in FlyBase (Supplementary Figure S3), strongly suggesting a true biological meaning. Fan and Kaguni [97] have shown that the fly Pol γ-β can bind an RNA primer with intra-strand base pairing, even if this molecule is not assembled in a primer-template structure that allows initiation of DNA synthesis. Pol γ-β is in fact a metazoan evolutionary novelty [30], with sequence and structural similarities to class IIa aminoacyl-tRNA synthetases [98], so binding to RNA is not necessarily surprising. However, its lack of correlational expression pattern with other mtDNA replisome factors and its correlations with factors related to mitochondrial transcription and transcript stability/degradation may suggest a dual role for Pol γ-β in mitochondria, assuming that the transcript levels shown here are at least partially reflected as proportional protein levels. The strong correlation with *Suv3* transcript levels (Figure 3 and Supplementary Figure S3) is perhaps the most striking one. This gene encodes a helicase that is able to unwind dsDNA, dsRNA and DNA/RNA heteroduplexes and is required for processing of polycistronic transcripts, removal of antisense RNA, and general messenger and transfer RNA stability in *Drosophila* mitochondria [99,100]. In the next section, we discuss the possible roles of other helicases in fly mtDNA maintenance.

We also analyzed relative transcript levels of *PolG1*, *mtDNA-helicase* and *mtSSB* from fly tissues represented in FlyAtlas 2 and modENCODE in pairwise comparisons, and verified they all correlate (Figure 3B and Supplementary Figure 3B), although PCA analysis indicates *mtSSB* levels are appreciably more distant from those of *PolG1* and *mtDNA-helicase* (Supplementary Figure S2). Considering that we have previously proposed that alterations in the stoichiometry of the mtDNA replisome, which would result from overexpression or depletion of one of its factors, could induce the formation of mtDNA deletions [23,26], we decided to test if the transcriptomic data could provide any insights into this matter. We analyzed the data using the absolute expression values reported in FlyAtlas 2 (in fragments per kilobase million, FPKM), once again making the assumption that these might correlate with protein levels inside mitochondria. We found that *PolG2* levels are consistently higher (∼5.8-fold on average, ±2.7) than those of *PolG1* across all tissues (Supplementary Table S3). This excess is well above that of the homotetrameric mtSSB (∼1.8-fold, ±0.6) (Supplementary Table S4), which is considered one of the most abundant mtDNA proteins [101,102]. It also indicates there would always be sufficient Pol γ-β to function in association with Pol γ-α to form the dimeric holoenzyme in a 1:1 configuration for mtDNA replication, in agreement with previously published data [103] and with our speculations on additional roles for the fly Pol γ-β in mitochondrial RNA metabolism. Our data also contrast with data from human tissues, which indicates 2- to 3-fold excess of Pol γ-α over Pol γ-β [43]. *PolG1* levels were higher than those of *PolG2* (∼62%) only in the ovaries, a tissue that also has elevated relative expression of almost all mtDNA maintenance genes (Figure 2) due to the high levels of mtDNA replication and mitochondrial biogenesis that occur during oogenesis [104,105]. The reasons why *PolG2* levels are lower in a tissue with high production of mitochondria and mtDNA are intriguing and warrant further investigation, but it is worth mentioning that the female germline is the only type of cells for which selection against mutated mtDNA molecules has been shown in *Drosophila* [106].

We also observed in our data another striking correlation between the absolute expression values of *mtDNA-helicase* and *PolG1*: the ratio between their transcript levels averaged at ∼1.2 (±0.48) (Supplementary Table S5). Supposing once again that transcript levels are directly reflected in polypeptide levels and that *Drosophila* Twinkle is homohexameric inside mitochondria, these numbers indicate a ∼5.8-fold (±2.3) excess of Pol γ holoenzyme over Twinkle, and that the latter is the limiting factor in mtDNA replication, at least in terms of fork progression of the mtDNA replisome in its ‘static view’. Interestingly, to our knowledge, Twinkle is the only fly mtDNA replisome factor whose overexpression increases mtDNA copy number [35,67,68], but it also leads to mtDNA deletions [23], indicating its levels might be controlled to determine a functional number of intact copies of mtDNA in each tissue. However, quite surprisingly, there appear to exist tissues in *Drosophila* in which Twinkle dysfunction or misexpression has no measurable phenotypic and biochemical effects [68]. We present new data on the matter and discuss the relevance of these findings in the next section. In summary, based solely on transcriptomic data analyses of fly tissues from the FlyAtlas 2 databank, we speculate that on average for each hexameric Twinkle, there are ∼5.4 dimeric Pol γ holoenzymes, ∼8.5 tetrameric mtSSBs, and ∼22 extra Pol γ-β molecules. We believe we present for the first time a rough estimate of the population of mtDNA replisome molecules in *Drosophila* mitochondria from a significant variety of tissues. Although this is potentially informative, it is not conclusive at any level and must be seen with extreme caution. Further analyses of the tissue-specific expression levels of the other mtDNA maintenance genes are required for a better understanding of the DNA and RNA metabolic processes involved, and extensive work is necessary for our predictions to be tested experimentally.

## Twinkle and the potential involvement of additional helicases

We have shown previously that ubiquitously expressed, defective versions of Twinkle cause lethality at the *Drosophila* larval stage but inducing their expression only in neurons or muscles surprisingly does not impact development [68]. The nervous system and the musculature are tissues with high demand for OXPHOS-driven ATP synthesis, and therefore mitochondrial dysfunction is expected to have phenotypic outcomes. To explore this further in the context of possible tissue-specific mtDNA maintenance processes, here we present preliminary analyses of Twinkle dysfunction in the adult fly nervous system and musculature, and a short perspective on possible roles of other helicases in *Drosophila* mtDNA replication. We used the *UAS*/*GAL4* system to overexpress wild-type Twinkle (WT) or the dominant-negative Twinkle K388A mutant [67] using the pan-neuronal *elavGAL4* and the muscle-specific *mhcGAL4* drivers. Overexpression was confirmed by immunoblotting using mitochondrial extracts from adult heads (where most of the central nervous system is located) and thoraces (where the indirect and direct flight muscles are found); once again it did not cause developmental failure when directed to these energy-demanding tissues (Supplementary Figure S4). K388 in the fly Twinkle is the equivalent of human Twinkle K421 and T7 gp4 K318, a highly conserved lysine residue in the Walker A motif of the C-terminal helicase domain of these enzymes, which is essential for ATP binding and hydrolysis, and thus for dsDNA unwinding at the replication fork [35,90,107,108]. One could say absence/replacement of this lysine is incompatible with cellular and organismal viability.

**Figure 4:**
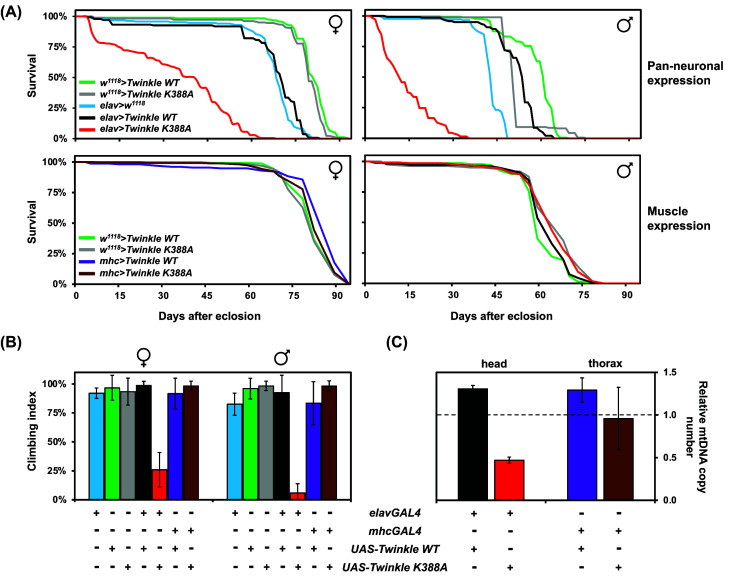
Twinkle dysfunction has distinct effects on the *Drosophila* nervous system and musculature Lifespan (**A**), climbing (**B**) and quantitative PCR (**C**) assays were performed as described in the Methods, using flies or fly tissue extracts as indicated. mtDNA copy number of Twinkle overexpressors in (**C**) was respectively normalized by the levels in each control *UAS-Twinkle* line, which were arbitrarily set to 1.0 (indicated with dashed line).

We next checked if adult Twinkle overexpressors had altered longevity, and verified a considerable decrease in medium and maximum lifespan of flies overexpressing Twinkle K388A in neurons (*elav>Twinkle K388A*) (Figure 4A, upper panels). We also observed that the transgenic construct *elavGAL4*, independent of *UAS-Twinkle*, reduced longevity in *Drosophila*, as both *elavGAL4* driver control (*elav>w^1118^*) and *elav>Twinkle WT* flies lived an average of 10 days less than controls without *elavGAL4*. We assume that the *GAL4* transgene in this driver line, which was inserted into the promoter region of the endogenous *elav* gene on the chromosome X [109,110], interferes with some functions of the gene, affecting adult longevity. We also performed climbing assays to verify if the decrease in longevity induced by Twinkle K388A is due to neurolocomotor problems in adult flies, and observed a severe inability of *elavGAL4>Twinkle K388A* flies to climb (Figure 4B). Some of the individuals were so severely affected that during the course of our experiment, they did not even try to climb (an ability known as negative geotaxis, quite natural to healthy adult flies), or fell backwards immediately after trying (data not shown). The phenotype was more pronounced in males.

Unlike the neuron-specific effects, neither Twinkle K388A nor WT overexpression in the muscles led to any detectable changes in longevity and locomotor ability of adult flies (Figure 4A, lower panels, and B). We then measured relative mtDNA copy number in head and thorax extracts, and observed that overexpression of Twinkle WT in neurons and in muscles caused an increase of ∼30% in mtDNA levels in both tissues (Figure 4C). This corroborates previously published data for mice [111,112], adult flies and cultured S2 cells [35,68], again suggesting that Twinkle is a limiting factor for mtDNA replication. Overexpression of Twinkle K388A in the nervous system dropped mtDNA copy number more than 50% (Figure 4C), which is consistent with a mitochondrial dysfunction leading to locomotor defects and reduced lifespan in the *elavGAL4>Twinkle K388A* adult flies. The musculature of *mhc>Twinkle K388A* individuals, on the other hand, showed no significant changes in mtDNA levels (Figure 4C), consistent with the idea that Twinkle K388A is neutral in muscle tissues of *Drosophila*, despite being expressed at very high levels (Supplementary Figure S4B). The adult thoracic musculature (which includes the muscles that control the flying and the negative geotaxis behaviors) of *D. melanogaster* is one of the most efficient tissues in OXPHOS-driven ATP production in the animal kingdom [113], with mitochondria occupying up to a third of the total volume of the cells [114]. Therefore, the absence of altered locomotor ability, longevity and mtDNA levels in *mhcGAL4>Twinkle K388A* flies is extremely intriguing. Our results suggest that although Twinkle is indispensable for mtDNA maintenance in *Drosophila* neurons, it may play no role at all in the adult musculature.

Because in whole adult fly samples the vast majority of isolated mitochondria (and therefore mtDNA molecules) come from the thoracic musculature, it is safe to assume that the main mode of mtDNA replication described for adult individuals ([17] and see above) is in fact the mode for this tissue. If that is indeed correct, it is puzzling that mtDNA maintenance has been described basically identically in S2 cells and in adult flight muscles, and yet a severely defective Twinkle enzyme causes a strong mtDNA depletion in the former [35] and no detectable phenotype in the latter (Figure 4). Note that the FlyAtlas 2 and modENCODE data in this case does not help us understand the possible tissue-specific roles of Twinkle in *Drosophila*, since expression levels of *mtDNA-helicase* are extremely low in adult heads, brains, eyes, and carcasses (Figure 2). The carcass, from which muscle transcriptomic data is derived, is particularly complicated to analyze as it is probably contaminated with the high levels of *mtDNA-helicase* transcripts from ovarian tissues in females (please refer to www.flyatlas2.org [94,95] for details on the nature of the carcass), artifactually suggesting sex-specific differences. New methods to analyze mtDNA replication intermediates must then be applied to solve this issue. We envision that these analyses from fly tissues of Twinkle overexpressors might reveal yet new mechanisms of mtDNA maintenance in animals, some of which may be independent of Twinkle. Expanding our knowledge on the diversity of nucleic acid metabolic processes in animal mitochondria will be quite an exciting research path to take in the near future.

In mammals, Twinkle is considered the only mitochondrial replicative helicase, but four other helicases have also been shown to localize to mitochondria (reviewed in [115]), all of which with homologs in flies. As mentioned above, the *Drosophila* Suv3 appears to be essential for mitochondrial RNA metabolism [99,100]. However, the fact that the human homologue unwinds dsDNA more efficiently than dsRNA and that knockout of the mouse homolog causes accumulation of mtDNA mutations [116] leads us to speculate a potential additional function for this enzyme. It could perhaps function as a replicative helicase, at least when Twinkle is absent or not required for mtDNA maintenance as we suggest here for the fly musculature. It is also possible that Suv3 functions as a mtDNA repair enzyme; in this case in *Drosophila*, the correlation between the expression levels of *Suv3* and *PolG2* would indicate that the putative excess of Pol γ-β molecules inside mitochondria are also for DNA repair purposes.

The *Drosophila* Pif1, Dna2 and RecQ4 helicases (encoded by the *Pif1*, *Dna2* and *RecQ4* genes, respectively), on the other hand, appear to lack mitochondrial targeting sequences (Supplementary Table S1). In addition, mutations in these genes cause no clear mitochondrial and/or mtDNA phenotype [117–121], unlike mutations in the human *DNA2* gene and ablation of the mouse *PIF1* gene, which are clearly associated with mitochondrial myopathy [122,123]. The transcriptomic data presented above again do not provide any hints to which of these might have a replicative function in the absence of Twinkle. Alternatively, it is possible that in the fly musculature Twinkle requires a partner factor for proper function, perhaps a helicase loader, although it has been suggested that the human Twinkle *in vitro* does not require such a partner for loading onto a closed circular mtDNA molecule [124]. More extensive coexpression analyses of the muscle transcriptomics, and mutant/RNAi/protein-protein interaction screenings are plausible experimental approaches for future investigations of this issue.

## Concluding remarks

The mechanisms of mtDNA maintenance in *Drosophila*, as in mammals, appear to be diverse, although much still remains to be revealed experimentally. Flies have been and will continue to be important models for our understanding of mtDNA maintenance in human and other animal cells, but the mechanistic differences among these organisms must be taken into consideration for good models to be established. This is especially true for the mtDNA repair processes, which probably occur substantially differently in *Drosophila* and human mitochondria due to the distinct selective pressures that lead to mitochondrial genomes with separate nucleotide composition biases. This is evidenced by the lack of many human mtDNA repair orthologues in flies, and by the fact that factors proven to be important in human mitochondria, such as Ogg1, appear to have insignificant impact on *Drosophila* mtDNA maintenance. On the other hand, the conservation of at least the main mode of mtDNA replication and of the mtDNA replisome and transcription genes between flies and mammals suggests an important correlation with the highly compact structure and the highly conserved gene content of animal mitochondrial genomes, which are unique among eukaryotes. Importantly, more than just considering ‘a’ mtDNA replication mode and ‘the’ mtDNA replication (or transcription) machinery, we believe these are dynamic in nature both in flies and mammals, and may have tissue specificity to accommodate the physiology of particular cell types. This was here exemplified by the distinct roles of the fly Twinkle, ‘the’ mitochondrial replicative helicase, whose dysfunction in the musculature apparently has no effect on tissue and organismal viability, whereas in the nervous system it leads to expected neurolocomotor defects. We hope this review can highlight some of the interesting paths investigators can take to help our community show how *Drosophila* may impact future mtDNA maintenance research.

## Methods

### *In silico* tissue-specific gene expression analyses

Genes important for mtDNA maintenance (Supplementary Table S2), and representative OXPHOS and other mitochondrial genes (see legend to Supplementary Figure S3 for FlyBase ID numbers) in *D. melanogaster* were used. Expression values (RNA-seq data) of these genes in different tissues in larva, pupa, and adult male and female were obtained from the FlyAtlas 2 (www.flyatlas2.org) [94,95] and the FlyBase (http://flybase.org/) [125] databases, as fragments per kilobase million (FPKM) and reads per kilobase million (RPKM), respectively. The data were normalized per tissue by the expression level of the housekeeping *eIF1A* gene (FlyBase ID FBgn0026250). A mean expression level among all genes of all tissues was calculated and used as a normalization factor across tissues to obtain the relative expression level of a gene per tissue, which was then converted to base 2 logarithm. Hierarchical cluster analyses were conducted using EXPANDER (http://acgt.cs.tau.ac.il/expander/) [126], with the complete linkage method and the Pearson’s correlation similarity measure. EXPANDER was also used for the principal component analyses. Pairwise correlations and linear regressions were performed using Microsoft Excel. R was used to perform the Kolmogorov–Smirnov test for normal distribution of the log transformed data and the Spearman’s ρ correlations, as indicated in the figure legends.

### *Drosophila* stock maintenance and phenotypic assays

All fly lines were backcrossed into the *w^1118^* background for at least six generations. The standard line *w^1118^*, the Twinkle lines *UAS-Twinkle WT/TM6B* and *UAS-Twinkle K388A* [67], and driver lines *myosin heavy chain-GAL4* (*mhcGAL4*) and *embryonic lethal abnormal vision^C155^-GAL4* (*elavGAL4*) were used. Flies were cultured on standard diet [127] at 25°C, with 12-h light/dark cycles and manually controlled humidity.

Fifteen virgin *UAS-Twinkle* females were crossed, per vial, with 5-7 *w^1118^* or *mhcGAL4* males to respectively generate progenies of uninduced controls or induced muscle-specific Twinkle overexpressors. For the pan-neuronal overexpressors, virgin *elavGAL4* females were crossed with *UAS-Twinkle* males. For the developmental assays, 60–90 eggs were monitored daily and kept until adult eclosion. Pupal viability was calculated as the mean ratio between the number of eclosed adults and the number of pupae per vial, with eight vials per experiment, and three biological replicates. For the longevity assays, 100–200 recently eclosed adult males and females were collected, kept separately in vials with 10–20 flies each, and transferred to new vials containing fresh food every 2–3 days, at which time dead flies were counted to generate a survival curve.

Climbing assays were conducted as described previously [128], with a few modifications. Forty-eighty adult females and males were collected on ice three days after eclosion. Groups of 10 flies were acclimated for 1 h at 25°C, and were transferred to transparent 19 cm-long climbing vials, which were then tapped on the bench 3–6 times for flies to fall to the bottom and start climbing. The climbing was recorded using a camera positioned 20 cm away from the vials and the videos were analyzed using the ImageJ software [129]. The percentage of flies that reached a 6 cm mark within 5 s was defined as climbing index.

### Immunoblotting

Approximately 40 thoraces of *mhc>Twinkle* males and 200 heads of *elav>Twinkle* males and females (5 day old) were homogenized in 1 ml of isolation buffer (200 mM sucrose, 5 mM Tris/HCl, 2 mM EGTA, 2 mM DTT, pH 7.4) on ice, and centrifuged at 4°C for 1 min at 200 ***g***_max_. The supernatant was recentrifuged at 4°C for 3 min at the same speed, and then for 10 min at 9000 ***g***_max_. The pellet was resuspended in 1 ml of isolation buffer, recentrifuged for 10 min at 9000 ***g**_max_*, and finally resuspended in 200 µl of isolation buffer to generate a crude mitochondrial fraction. Protein concentration was estimated via absorbance at 280 nm using NanoDrop 2000 (Thermo Fisher Scientific, U.S.A.). Respectively, 80 and 40 µg of extracts from thorax or head mitochondria were mixed with 4× Laemmli buffer (10% SDS, 50% glycerol, 25% 2-mercaptoethanol, 0.02% bromophenol blue, and 0.3125 M Tris-HCl, pH 6.8), and denatured for 5 min at 95°C. Samples were resolved by SDS-PAGE on 12% polyacrylamide gels at 80 V for 3 h. Proteins were transferred to a nitrocellulose membrane using a semi-dry gel transfer system (Major Science, U.S.A.) for 7 min at room temperature. Membranes were initially blocked overnight at 4°C in PBS/Tween 20 (8 mM Na_2_HPO_4_, 2 mM KH_2_PO_4_, 150 mM NaCl, 30 mM KCl, 0.05% Tween 20, pH 7.4) containing 5% dried nonfat milk. Afterward, the membranes containing samples of the thorax mitochondrial extracts were incubated with a mixture of the primary antibodies (1:10,000 dilution of rabbit polyclonal anti-Dm helicase [35] and 1:12,000 dilution of mouse monoclonal anti-PDH E1α (Abcam, U.K.) in PBS/Tween 20 containing 1% dried nonfat milk) for 1 h 30 min at room temperature, followed by three 30 min washes with PBS/Tween 20 and subsequent incubation with a mixture with the secondary antibodies (HRP-conjugated anti-rabbit and -mouse IgGs (Bio-Rad, U.S.A.) and washings, as described above. Membranes were incubated with Immun-Star HRP luminol detection system (Bio-Rad, U.S.A.) and chemiluminescence signals were detected using the ChemiDoc Imaging System (Bio-Rad, U.S.A.). The membranes containing samples of the head mitochondrial extracts were first treated with the primary anti-Dm helicase antibody and the secondary HRP-conjugated anti-rabbit IgG, signals were detected as described above, and the membranes were stripped with a solution of 200 mM glycine, 0.1% SDS, 1% Tween 20, pH 2.2, for 30 min at room temperature. The membranes were then re-blocked, treated with the primary mouse monoclonal anti-ATP5A antibody (1:10,000 dilution, Abcam, U.K.) and the secondary HRP-conjugated anti-mouse IgG, and processed as described above.

### Nucleic acid extraction and quantitative PCR

Total DNA was extracted from 10 heads and 3 thoraces of 7 day old *elav>Twinkle* and *mhc>Twinkle* adult males, respectively. Tissues were homogenized in 500 µl of lysis buffer (75 mM NaCl, 25 mM EDTA, 25 mM HEPES, pH 7.5) on ice, followed by addition of 50 µl of 10% SDS and 10 μl of proteinase K (20 mg/ml) and incubation for 1 h at 37°C. Nucleic acids were extracted by adding 500 µl of phenol:chloroform:isoamyl alcohol (25:14:1), mixing by inversion four times, incubating on ice for 1 min, centrifuging at 2000 ***g**_max_* for 5 min at 4°C, recovering the aqueous phase, and repeating the procedure once. Nucleic acids were precipitated by addition of 80 μl of 10 M ammonium acetate and 1 ml of ice-cold 100% ethanol, and overnight incubation at −20°C. Samples were centrifuged at 16,000 ***g**_max_* for 30 min at 4°C, and washed with ice-cold 70% ethanol. After drying in lyophilizer for 15 min, nucleic acids were resuspended in 30 μl of TE buffer (10 mM Tris/HCl, 0.1 mM EDTA, pH 7.5), and DNA concentration was estimated by absorbance at 260 nm using NanoDrop 2000 (Thermo Fisher Scientific, U.S.A.). To estimate the relative mtDNA copy number, 15 ng of total DNA were used as template in quantitative amplification reactions of a fragment of the mitochondrial *16S rRNA* gene (primers 5′-TCGTCCAACCATTCATTCCA-3′ and 5′-TGGCCGCAGTATTTTGACTG-3′), and a fragment of the single-copy nuclear *RpL32* gene (primers 5′-AGGCCCAAGATCGTGAAAGAA-3′ and 5′-TGTGCACCAGGAACTTCTTGAA-3′), as described previously [68]. Reactions were performed using the SYBR Green JumpStart™ Taq ReadyMix™ Kit (Sigma-Aldrich, DE) in the StepOnePlus™ Real-Time PCR System (Thermo Fisher Scientific, U.S.A.), under the manufacturer’s recommended conditions: 95°C for 2 min, 40 cycles of 95°C for 3 s and 60°C for 30 s, followed by melting curve. The ΔΔ*C*_T_ values were calculated by comparing the ratios between the mitochondrial and nuclear target genes.

## Supplementary Material

Supplementary Figures S1-S4 and Tables S1-S5

## Data Availability

Processed and raw data of the analyses presented here can be obtained from the corresponding author upon request.

## Abbreviations

2D-NAGE: 2D native gel electrophoresis
BER: base excision repair
OXPHOS: oxidative phosphorylation
PCA: principal component analysis
Pol γ: DNA polymerase γ
ROS: reactive oxygen species
