# Supplementary material for: Mitochondrial DNA maintenance in Drosophila melanogaster
Source: Biosci Rep. 2022 Nov 10;42(11):BSR20211693. doi: 10.1042/BSR20211693 (PMC9653094; doi:10.1042/BSR20211693)
Supplement: Supplementary Figures S1-S4 and Tables S1-S5 [file BSR-2021-1693C_supp.pdf]

Supplementary Material

Mitochondrial DNA maintenance in *Drosophila melanogaster*

Ana P. C. Rodrigues, Audrey C. Novaes, Grzegorz L. Ciesielski & Marcos T. Oliveira

Supplementary Tables

Supplementary Table 1. List of DNA repair proteins found in human mitochondria* and their homologues in *D. melanogaster*.

Product of the human gene (HGNC symbol)	*Drosophila* homologue	
	FlyBase symbol	FlyBase ID
ALKBH1	*AlkB*	FBgn0065035
APEX1	*Rrp1*	FBgn0004584
APTX	*CG5316*	FBgn0038704
CDK1	*Cdk1*	FBgn0004106
DNA2	*Dna2*	FBgn0288690
EXD2	*Exd2*	FBgn0037901
FEN1	*Fen1*	FBgn0025832
FH	*Fum1*[£]	FBgn0286222
	Fum2	FBgn0029890
LIG3	*DNAlig3*[£]	FBgn0286075
MLH1	*Mlh1*	FBgn0011659
OGG1	*Ogg1*	FBgn0027864
PARK7	*DJ-1β*	FBgn0039802
PARP1	*Parp*	FBgn0010247
PIF1	*Pif1*	FBgn0031540
PNKP	*PNKP*	FBgn0037578
POLDIP2	*POLDIP2*[£]	FBgn0037329
POLG	*PolG1*[#]	FBgn0004406
POLG2	*PolG2*[#]	FBgn0004407
RAD51	*spn-A*	FBgn0003479
RAD51C	*spn-D*	FBgn0003482
RECQL4	*RecQ4*	FBgn0040290
RPS3	*RpS3*	FBgn0002622
RUVBL2	*rept*	FBgn0040075
SLC30A9	*ZnT49B*[£]	FBgn0033762
UBE2N	*CG3473*	FBgn0028913
VCP	*TER94*	FBgn0286784
XRCC3	*spn-B*	FBgn0003480
APEX2	-	-
BRCA1	-	-
ERCC6L2	-	-
ERCC8	-	-
FANCG	-	-
FOXM1	-	-
MGME1	-	-
MPG	-	-
MUTYH	-	-
NUDT1	-	-

PRIMPOL	-	-
TIGAR	-	-
UNG	-	-

* according to (1).

£ *in silico* prediction of at least one polypeptide product containing a mitochondrial targeting sequence, based on TargetP 2.0 analyses (2).

mitochondrial localization empirically confirmed (3).

- indicates no gene with significant sequence similarity was found to characterize a true homologue with possible conserved function.

Supplementary Table 2. List of mitochondrial DNA maintenance genes of *D. melanogaster* for which tissue-specific transcript levels were used in this work.

Gene			Human homologue
Symbol	Name	FlyBase ID	
DNA2	DNA replication helicase/nuclease 2	FBgn0288690	*DNA2*
DNAlig3	DNA ligase 3	FBgn0286075	*LIG3*
Fen1	flap endonuclease 1	FBgn0025832	*FEN1*
mtDNA-helicase	mitochondrial DNA helicase	FBgn0032154	*TWNK*
mTerf3	mitochondrial transcription termination factor 3	FBgn0037008	*MTERF3*
mTerf5	mitochondrial transcription termination factor 5	FBgn0038584	-
mtSSB	mitochondrial single stranded DNA-binding protein	FBgn0010438	*SSBP1*
mtTFB1	mitochondrial transcription factor B1	FBgn0261381	*TFB1M*
mtTFB2	mitochondrial transcription factor B2	FBgn0037778	*TFB2M*
mTTF	mitochondrial transcription termination factor	FBgn0028530	-
Pif1	Pif1 DNA helicase	FBgn0031540	*PIF1*
PolG1	DNA polymerase gamma subunit 1	FBgn0004406	*POLG*
PolG2	DNA polymerase gamma subunit 2	FBgn0004407	*POLG2*
PolrMT	RNA polymerase mitochondrial	FBgn0261938	*POLRMT*
rnh1	ribonuclease H1	FBgn0023171	*RNASEH1*
Suv3	Suv3 helicase	FBgn0037232	*SUPV3L1*
TFAM	mitochondrial transcription factor A	FBgn0038805	*TFAM*
Top1	topoisomerase 1	FBgn0004924	*TOP1/ TOP1MT*
Top2	topoisomerase 2	FBgn0284220	*TOP2A/ TOP2B*
Top3α	topoisomerase 3α	FBgn0040268	*TOP3A*
Top3β	topoisomerase 3β	FBgn0026015	*TOP3B*

Supplementary Table 3. Absolute transcript levels and estimated protein ratios[$] of *PolG1* and *PolG2* across *Drosophila* tissues.

Tissues	*PolG1*[£]	*PolG2*	*PolG2* / *PolG1*	Estimated Pol γ-β / Pol γ-α
AM* accessory glands	0.88	6.80	7.73	7.73
AF brain	1.08	9.68	8.96	8.96
AM brain	1.27	9.75	7.68	7.68
L brain	2.45	10.02	4.09	4.09
AF carcass	1.93	6.71	3.48	3.48
AM carcass	1.19	7.31	6.14	6.14
L carcass	1.11	7.23	6.51	6.51
AF crop	1.25	8.28	6.62	6.62
AM crop	1.48	7.62	5.15	5.15
AF eye	1.84	9.46	5.14	5.14
AM eye	1.56	9.06	5.81	5.81
AF fat body	1.42	2.73	1.92	1.92
AM fat body	0.64	5.93	9.27	9.27
L fat body	1.90	9.54	5.02	5.02
L Garland cells	1.26	6.94	5.51	5.51
AF head	1.34	5.87	4.38	4.38
AM head	0.91	5.97	6.56	6.56
AF heart	3.83	6.58	1.72	1.72
AM heart	1.07	10.90	10.19	10.19
AF hindgut	1.20	9.35	7.79	7.79
AM hindgut	1.05	8.83	8.41	8.41
L hindgut	1.49	6.47	4.34	4.34
AF Malpighian tubules	1.46	6.65	4.55	4.55
AM Malpighian tubules	1.60	6.25	3.91	3.91
L Malpighian tubules	1.57	6.18	3.94	3.94
AF midgut	1.27	5.03	3.96	3.96
AM midgut	1.66	3.32	2.00	2.00
L midgut	1.25	5.23	4.18	4.18
AF ovary	6.13	3.78	0.62	0.62
AF rectal pad	0.84	7.62	9.07	9.07
AM rectal pad	0.89	6.96	7.82	7.82
AF salivary gland	2.04	4.58	2.25	2.25
AM salivary gland	1.05	7.23	6.89	6.89
L salivary gland	1.55	8.96	5.78	5.78
AF virgin spermatheca	0.84	3.54	4.21	4.21
AF mated spermatheca	0.86	4.89	5.69	5.69

AF TAG	1.15	13.45	11.70	11.70
AM TAG	1.06	14.41	13.59	13.59
AM testis	4.39	10.00	2.28	2.28
L trachea	1.93	5.93	3.07	3.07
average (+/- sd)	-	-	5.70 (+/- 2.81)	5.70 (+/- 2.81)
average – ovary	-	-	5.83 (+/- 2.73)	5.83 (+/- 2.73)

$ estimations were based on the assumption that protein levels inside mitochondria are proportional to the transcript levels obtained from transcriptomic data. This information must be used with caution.

* abbreviations: AM, adult male; AF, adult female; L, larval; TAG, thoracicoabdominal ganglion; sd, standard deviation.

£ transcript data is provided in FPKM, according to FlyAtlas 2 (4).

Supplementary Table 4. Absolute transcript levels and estimated protein ratios[$] of *PolG1* and *mtSSB* across *Drosophila* tissues.

Tissues	*PolG1*[£]	*mtSSB*	*mtSSB* / *PolG1*	mtSSB[#] / Pol γ
AM* accessory glands	0.88	7.82	8.89	2.22
AF brain	1.08	5.99	5.55	1.39
AM brain	1.27	6.48	5.10	1.28
L brain	2.45	20.69	8.44	2.11
AF carcass	1.93	11.72	6.07	1.52
AM carcass	1.19	6.59	5.54	1.38
L carcass	1.11	11.00	9.91	2.48
AF crop	1.25	14.04	11.23	2.81
AM crop	1.48	9.91	6.70	1.67
AF eye	1.84	6.69	3.64	0.91
AM eye	1.56	4.62	2.96	0.74
AF fat body	1.42	7.09	4.99	1.25
AM fat body	0.64	6.69	10.45	2.61
L fat body	1.90	19.60	10.32	2.58
L Garland cells	1.26	5.35	4.25	1.06
AF head	1.34	10.80	8.06	2.01
AM head	0.91	9.23	10.14	2.54
AF heart	3.83	12.67	3.31	0.83
AM heart	1.07	8.92	8.34	2.08
AF hindgut	1.20	8.16	6.80	1.70
AM hindgut	1.05	7.25	6.90	1.73
L hindgut	1.49	8.06	5.41	1.35
AF Malpighian tubules	1.46	9.68	6.63	1.66
AM Malpighian tubules	1.60	6.09	3.81	0.95
L Malpighian tubules	1.57	5.55	3.54	0.88
AF midgut	1.27	8.28	6.52	1.63
AM midgut	1.66	8.07	4.86	1.22
L midgut	1.25	7.31	5.85	1.46
AF ovary	6.13	70.81	11.55	2.89
AF rectal pad	0.84	7.57	9.01	2.25
AM rectal pad	0.89	6.07	6.82	1.71
AF salivary gland	2.04	14.56	7.14	1.78
AM salivary gland	1.05	7.45	7.10	1.77
L salivary gland	1.55	17.46	11.26	2.82
AF virgin spermatheca	0.84	5.56	6.62	1.65
AF mated spermatheca	0.86	4.87	5.66	1.42

AF TAG	1.15	7.67	6.67	1.67
AM TAG	1.06	7.12	6.72	1.68
AM testis	4.39	11.18	2.55	0.64
L trachea	1.93	17.46	9.05	2.26
average (+/- sd)	-	-	6.86 (+/- 2.42)	1.71 (+/- 0.61)
average – testis	-	-	6.97 (+/- 2.35)	1.74 (+/- 0.59)
average – testis and eye	-	-	7.17 (+/- 2.24)	1.79 (+/- 0.56)

[$] estimations were based on the assumption that protein levels inside mitochondria are proportional to the transcript levels obtained from transcriptomic data. This information must be used with caution.

* abbreviations: AM, adult male; AF, adult female; L, larval; TAG, thoracicoabdominal ganglion; sd, standard deviation.

[£] transcript data is provided in FPKM, according to FlyAtlas 2 (4).

[#] considering functional mtSSB as a homotetrameric protein.

Supplementary Table 5. Absolute transcript levels and estimated protein ratios[$] of *PolG1* and *mtDNA-helicase* across *Drosophila* tissues.

Tissues	*PolG1*[£]	*mtDNA-helicase*	*mtDNA-helicase* / *PolG1*	Estimated Pol γ / Twinkle[#]
AM* accessory glands	0.88	2.47	2.81	2.14
AF brain	1.08	0.76	0.70	8.53
AM brain	1.27	0.77	0.61	9.90
L brain	2.45	3.20	1.31	4.59
AF carcass	1.93	2.78	1.44	4.17
AM carcass	1.19	1.45	1.22	4.92
L carcass	1.11	1.28	1.15	5.20
AF crop	1.25	1.48	1.18	5.07
AM crop	1.48	1.37	0.93	6.48
AF eye	1.84	1.27	0.69	8.69
AM eye	1.56	0.90	0.58	10.40
AF fat body	1.42	2.43	1.71	3.51
AM fat body	0.64	1.66	2.59	2.31
L fat body	1.90	1.28	0.67	8.91
L Garland cells	1.26	1.43	1.13	5.29
AF head	1.34	1.04	0.78	7.73
AM head	0.91	0.56	0.62	9.75
AF heart	3.83	3.85	1.01	5.97
AM heart	1.07	2.25	2.10	2.85
AF hindgut	1.20	2.25	1.88	3.20
AM hindgut	1.05	2.16	2.06	2.92
L hindgut	1.49	1.09	0.73	8.20
AF Malpighian tubules	1.46	2.17	1.49	4.04
AM Malpighian tubules	1.60	2.16	1.35	4.44
L Malpighian tubules	1.57	0.89	0.57	10.58
AF midgut	1.27	1.75	1.38	4.35
AM midgut	1.66	1.73	1.04	5.76
L midgut	1.25	1.15	0.92	6.52
AF ovary	6.13	6.79	1.11	5.42
AF rectal pad	0.84	1.24	1.48	4.06
AM rectal pad	0.89	1.13	1.27	4.73
AF salivary gland	2.04	2.72	1.33	4.50
AM salivary gland	1.05	2.00	1.90	3.15
L salivary gland	1.55	1.85	1.19	5.03
AF virgin spermatheca	0.84	0.70	0.83	7.20

AF mated spermatheca	0.86	1.40	1.63	3.69
AF TAG	1.15	0.94	0.82	7.34
AM TAG	1.06	0.92	0.87	6.91
AM testis	4.39	0.73	0.17	36.08
L trachea	1.93	2.43	1.26	4.77
average (+/- sd)	-	-	1.21 (+/- 0.56)	6.48 (+/- 5.32)
average – testis	-	-	1.24 (+/- 0.54)	5.72 (+/- 2.32)
average – testis and AG	-	-	1.20 (+/- 0.48)	5.82 (+/- 2.27)

[$] estimations were based on the assumption that protein levels inside mitochondria are proportional to the transcript levels obtained from transcriptomic data. This information must be used with caution.

* abbreviations: AM, adult male; AF, adult female; L, larval; TAG, thoracicoabdominal ganglion; sd, standard deviation; AG, accessory gland.

[£] transcript data is provided in FPKM, according to FlyAtlas 2 (4).

[#] considering functional Twinkle as a homohexameric protein.

Supplementary Figure Legends

Supplementary Figure 1. Hierarchical cluster analysis of the relative expression levels of *Drosophila* mtDNA maintenance and other mitochondrial genes across different fly tissues. Transcript levels were obtained from FlyAtlas 2 (4), processed as described in the Methods, and shown in the heatmap as $\log_2$(fold change). The analysis was performed using EXPANDER (5). L, AM and AF indicate larval, adult male and female, respectively. The tissues sampled were as described in the legend to Figure 2. The FlyBase ID of group 3 genes are: *ATPsynβ*, FBgn0010217; *ATPsynγ*, FBgn0020235; *COX5A*, FBgn0019624; *COX7A*, FBgn0040529; *Cyt-c-p*, FBgn0284248; *kdn*, FBgn0261955; *ND-20*, FBgn0030718; *ND-23*, FBgn0017567; *ND-30*, FBgn0266582; *ox*, FBgn0011227; *porin*, FBgn0004363; *RFeSP*, FBgn0021906; and *sesB*, FBgn0003360.

Supplementary Figure 2. Principal component analysis of the tissue-specific transcript data from *Drosophila* mtDNA maintenance genes. Transcript levels were obtained from FlyAtlas 2 (4), processed as described in the Methods, and analyzed using EXPANDER (5).

Supplementary Figure 3. *PolG2* expression across *Drosophila* tissues from the modENCODE project also correlates with mitochondrial RNA metabolism, and not mtDNA replisome genes. Lack of significant correlations between *PolG2* transcript levels and those of the indicated mtDNA replisome genes is shown in **A**. Significant correlations among the transcript levels of the other mtDNA replisome genes, and between *PolG2* transcript levels and those of the indicated mitochondrial RNA metabolism genes are shown, respectively, in **B** and **C**. Transcript

levels were obtained from modENCODE (http://www.modencode.org/, (6)) and were processed as described in the Methods. The R^2, Pearson's r and the significance F values were obtained from linear regression analyses performed using Microsoft Excel. Because the log converted expression data of *PolrMT* was not normally distributed, Spearman's ρ was also calculated for the pairwise comparison with *PolG2* and is shown in the graph. Tissues from which expression levels were calculated were: accessory gland from 4-day old mated adult male; carcass from wandering L3 larva, 1-, 4- and 20-day old adult; central nervous system from L3 larva and P8 pupa; digestive system from wandering L3 larva, 1-, 4- and 20-day old adult; fat body, wandering L3 larva, white prepupa and P8 pupa; head from 1-, 4- and 20-day old virgin adult female; head from 1-, 4- and 20-day old mated adult female; head, 1-, 4- and 20-day old mated adult male; imaginal disc from wandering L3 larva; ovary from 4-day old virgin and mated adult female; salivary gland from wandering L3 larva and white prepupa; testis from 4-day old mated adult male.

Supplementary Figure 4. Tissue-specific overexpression of Twinkle in flies. Pan-neuronal and muscle induction was respectively achieved as described in the Methods using the driver lines *elavGAL4* and *mhcGAL4*, and was confirmed via immunoblotting in crude mitochondrial extracts from adult heads (**A**) and thoraces (**B**) using the anti-Dm helicase antibody (7) to detect Twinkle. Levels of the alpha subunit of the ATP synthase complex (ATP5A) and the E1alpha subunit of the pyruvate dehydrogenase complex (PDH E1α) were respectively used as loading controls in **A** and **B**. "cross reaction" in **B** indicates an unspecific signal from the anti-Dm helicase antibody detected in extracts from thorax or whole adult mitochondria (8,9). MW, molecular weight marker Precision Plus Protein™ Standards (Bio-Rad, USA). **C**, pupal viability

was calculated as the average ratio between the number of eclosed adult flies and the number of pupae per vial, as described in the Methods.

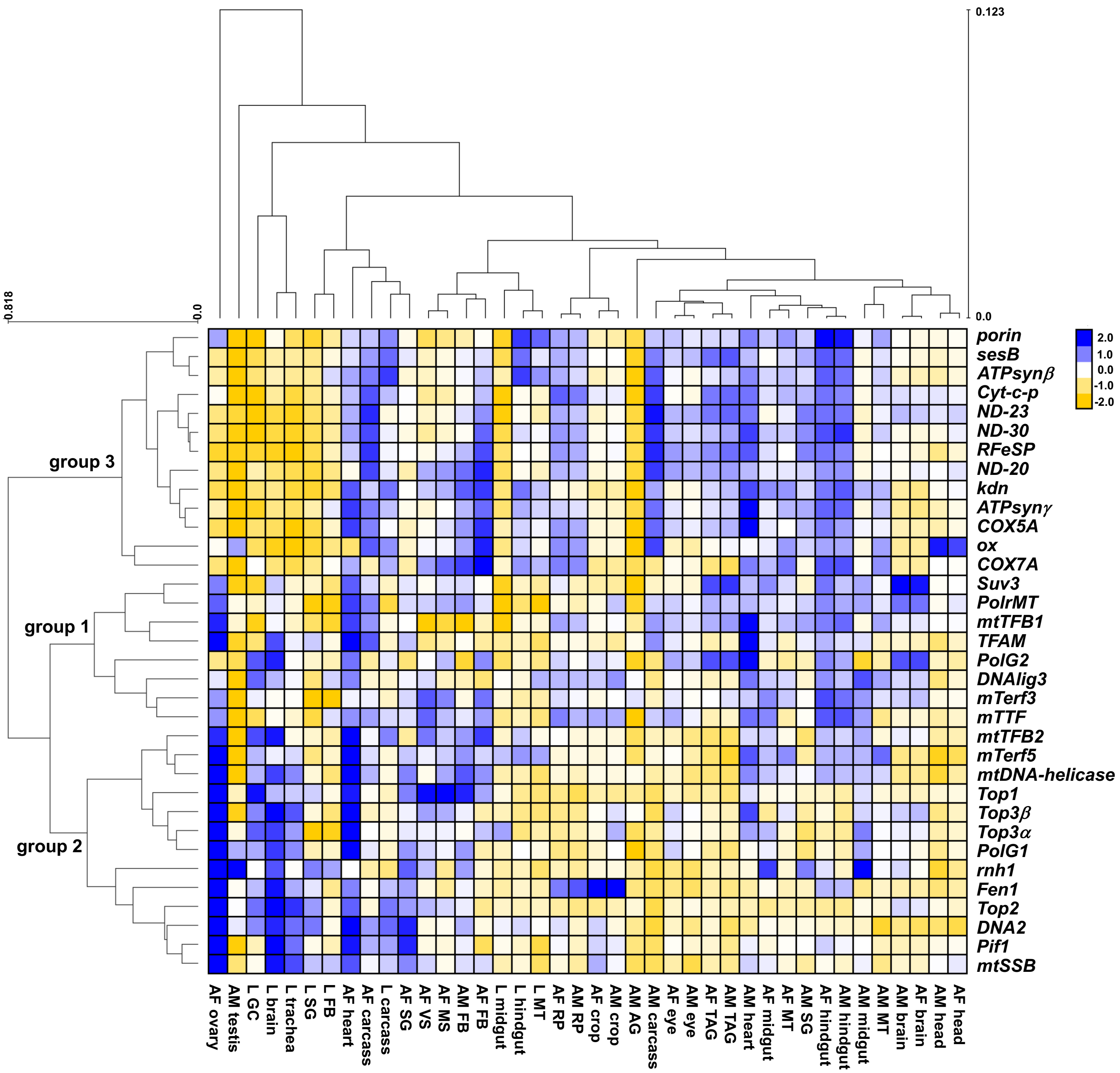

Supplementary Figure 1.

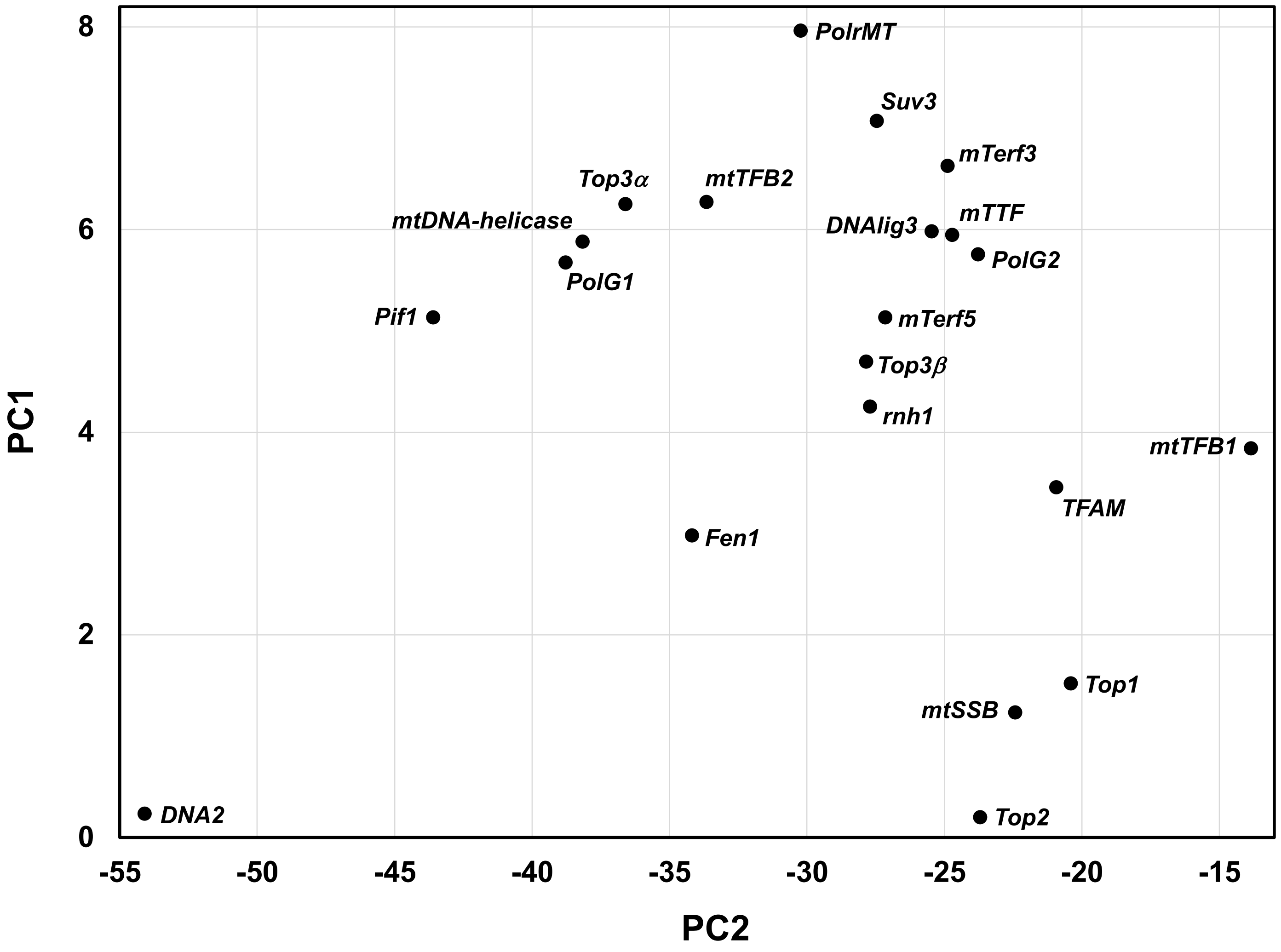

Supplementary Figure 2.

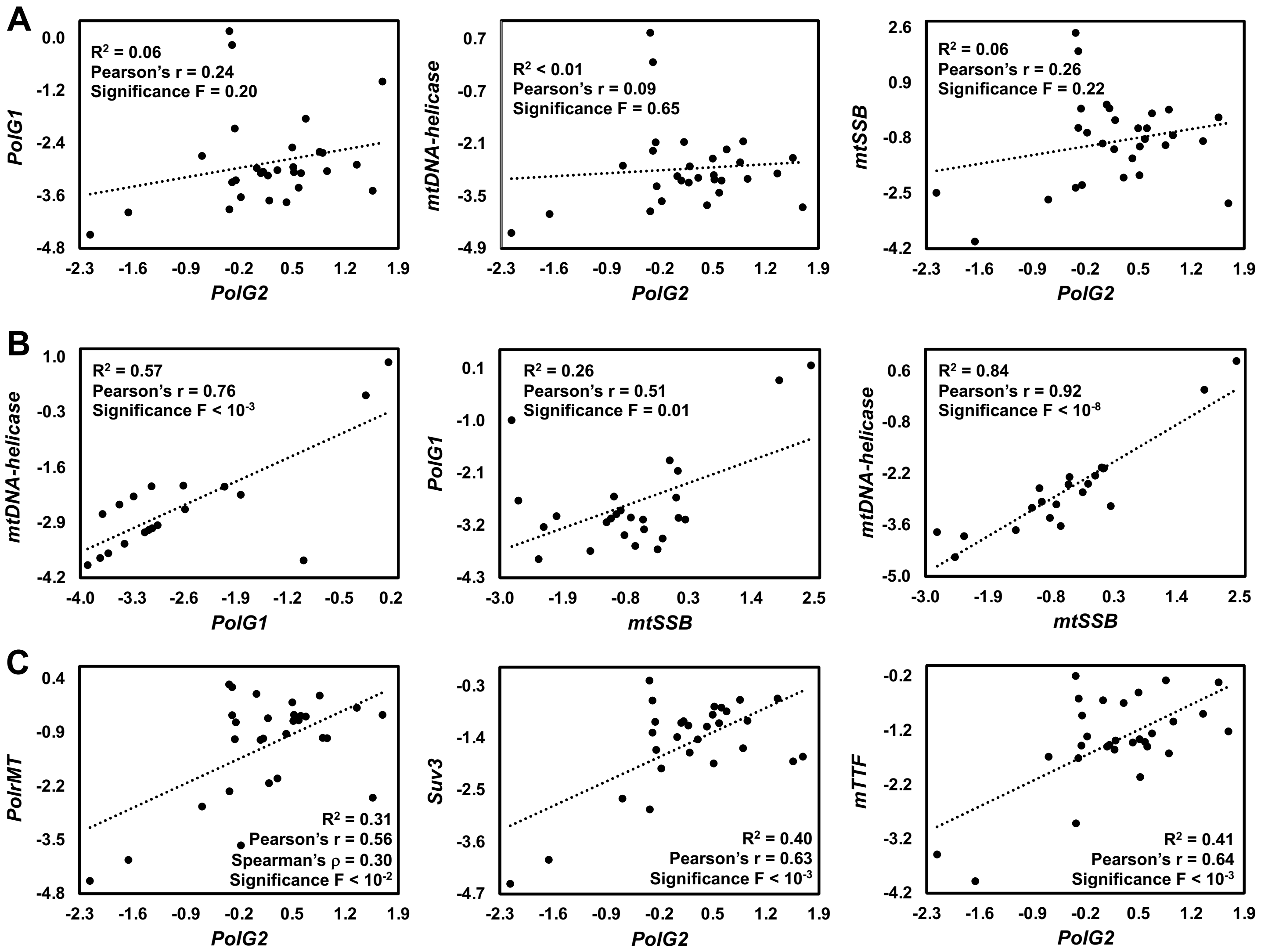

Supplementary Figure 3.

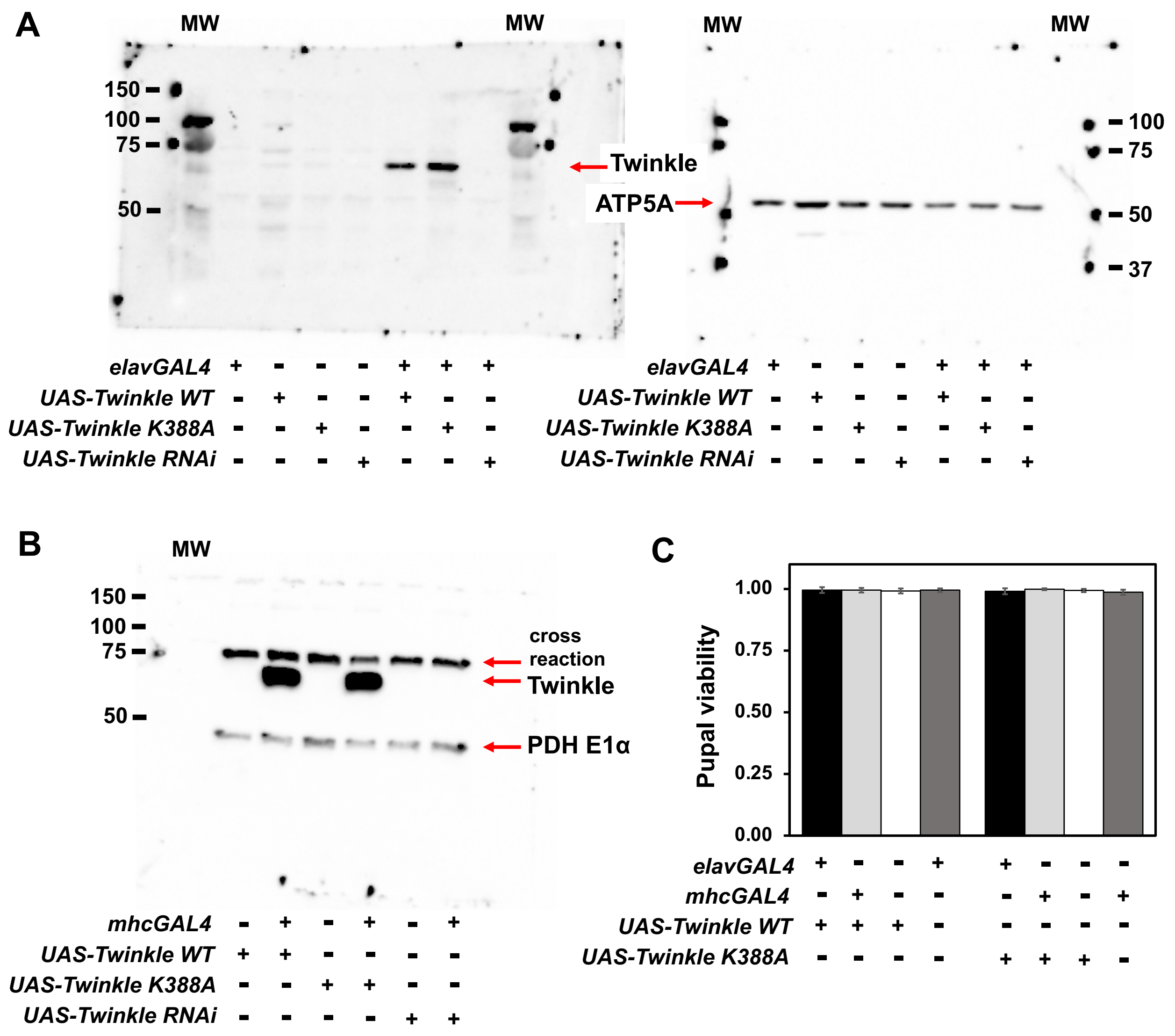

Supplementary Figure 4.

References

1. Allkanjari K, Baldock RA. Beyond base excision repair: An evolving picture of mitochondrial DNA repair. Biosci Rep. 2021;41(10):1–14. Available from: https://doi.org/10.1042/BSR20211320.

2. Armenteros JJA, Salvatore M, Emanuelsson O, Winther O, Von Heijne G, Elofsson A, et al. Detecting sequence signals in targeting peptides using deep learning. Life Sci Alliance. 2019;2(5):1–14. Available from: https://doi.org/10.26508/lsa.201900429.

3. Wernette CM, Kaguni LS. A mitochondrial DNA polymerase from embryos of *Drosophila melanogaster*. Purification, subunit structure, and partial characterization. J Biol Chem. 1986;261(31):14764–70. Available from: https://doi.org/10.1016/S0021-9258(18)66938-8.

4. Krause SA, Overend G, Dow JAT, Leader DP. FlyAtlas 2 in 2022: Enhancements to the *Drosophila melanogaster* expression atlas. Nucleic Acids Res. 2022;50(D1):D1010–5. Available from: https://doi.org/10.1093/nar/gkab971.

5. Hait TA, Maron-Katz A, Sagir D, Amar D, Ulitsky I, Linhart C, et al. The EXPANDER Integrated Platform for Transcriptome Analysis. J Mol Biol. 2019;431(13):2398–406. Available from: https://doi.org/10.1016/j.jmb.2019.05.013.

6. Roy S, Ernst J, Kharchenko P V, Kheradpour P, Negre N, et al. Identification of Functional Elements and Regulatory Circuits by *Drosophila* modENCODE. Science. 2011;330(6012):1787–97. Available from: https://doi.org/10.1126/science.1198374.

7. Matsushima Y, Kaguni LS. Differential Phenotypes of Active Site and Human Autosomal Dominant Progressive External Ophthalmoplegia Mutations in *Drosophila* Mitochondrial DNA Helicase Expressed in Schneider Cells. J Biol Chem. 2007;282(13):9436–9444. Available from: https://doi.org/10.1074/jbc.M610550200.

8. Sanchez-Martinez A, Calleja M, Peralta S, Matsushima Y, Hernandez-Sierra R, Whitworth AJ, et al. Modeling Pathogenic Mutations of Human Twinkle in *Drosophila* Suggests an Apoptosis Role in Response to Mitochondrial Defects. PLoS One. 2012;7(8):1–11. Available from: https://doi.org/10.1371/journal.pone.0043954.

9. Rodrigues APC, Camargo AF, Andjelković A, Jacobs HT, Oliveira MT. Developmental arrest in *Drosophila melanogaster* caused by mitochondrial DNA replication defects cannot be rescued by the alternative oxidase. Sci Rep. 2018;8(1):1–10. Available from: https://doi.org/10.1038/s41598-018-29150-x.
